# Dissecting the *Mycobacterium bovis* BCG Response to Macrophage Infection to Help Prioritize Targets for Anti-Tuberculosis Drug and Vaccine Discovery

**DOI:** 10.3390/vaccines10010113

**Published:** 2022-01-13

**Authors:** Jamie Medley, Aaron Goff, Paulo J. G. Bettencourt, Madelaine Dare, Liam Cole, Daire Cantillon, Simon J. Waddell

**Affiliations:** 1 Global Health and Infection, Brighton and Sussex Medical School, University of Sussex, Brighton BN1 9PX, UK; J.Medley@bsms.ac.uk (J.M.); A.Goff@bsms.ac.uk (A.G.); M.Dare1@uni.bsms.ac.uk (M.D.); Liam.cole@ggc.Scot.nhs.uk (L.C.); Daire.Cantillon@lstmed.ac.uk (D.C.); 2 Faculty of Medicine, Catholic University of Portugal, 1649-023 Lisbon, Portugal; pbettencourt@ucp.pt; 3 Center for Interdisciplinary Research in Health, Catholic University of Portugal, 1649-023 Lisbon, Portugal

**Keywords:** tuberculosis, *Mycobacterium*, macrophage, host-pathogen interactions, transcriptomics, BCG, RNAseq

## Abstract

New strategies are required to reduce the worldwide burden of tuberculosis. Intracellular survival and replication of *Mycobacterium tuberculosis* after macrophage phagocytosis is a fundamental step in the complex host–pathogen interactions that lead to granuloma formation and disease. Greater understanding of how the bacterium survives and thrives in these environments will inform novel drug and vaccine discovery programs. Here, we use in-depth RNA sequencing of *Mycobacterium bovis* BCG from human THP-1 macrophages to describe the mycobacterial adaptations to the intracellular environment. We identify 329 significantly differentially regulated genes, highlighting cholesterol catabolism, the methylcitrate cycle and iron homeostasis as important for mycobacteria inside macrophages. Examination of multi-functional gene families revealed that 35 PE/PPE genes and five cytochrome P450 genes were upregulated 24 h after infection, highlighting pathways of potential significance. Comparison of the intracellular transcriptome to gene essentiality and immunogenicity studies identified 15 potential targets that are both required for intracellular survival and induced on infection, and eight upregulated genes that have been demonstrated to be immunogenic in TB patients. Further insight into these new and established targets will support drug and vaccine development efforts.

## 1. Introduction

*Mycobacterium tuberculosis*, the cause of tuberculosis (TB), is estimated to have killed 1.5 million people in 2020 and is the leading cause of death by a bacterial agent, despite being a treatable disease. The disease burden is not distributed evenly, as 86% of those who fall sick with TB are in 30 countries, and 47% of all cases face catastrophic costs (>10% household income) to access treatment [1]. Progress has been hindered by the SARS-CoV-2 pandemic, with reduced access to medical support causing a drop in newly identified cases, an increase in deaths, and a reduction in treatment provision [1].

TB vaccination programs use the Bacille Calmette–Guerin (BCG) vaccine created from an attenuated culture of *Mycobacterium bovis* approximately 100 years ago [2]. Meta-analysis indicates that BCG vaccination significantly reduces risk of TB by 50%, though protection against death, TB meningitis and disseminated disease is higher [3]. There is large inter-study variation from 0–80% efficacy, likely due to a combination of factors including pre-exposure to environmental mycobacteria [4]. In the search for new and more-effective vaccines, the development pipeline requires constant replenishment of target antigens expressed by *M. tuberculosis* during infection [5].

Standard chemotherapy for drug-sensitive TB is a minimum 6 months of a combination of isoniazid, rifampicin, pyrazinamide and ethambutol, although recent evidence suggests that this might be safely reduced to 4 months with the substitution of rifapentine for rifampicin and moxifloxacin for ethambutol [6]. In addition to long treatment times and the disruption of TB clinical services by SARS-CoV-2, the emergence of drug resistance threatens TB control programs worldwide. The World Health Organization estimates that 3–4% of new TB cases and 18–21% of previously treated cases are resistant to one or more first-line antibiotics (rifampicin +/− isoniazid) [1]. Current first- and second-line anti-TB drugs inhibit a limited number of *M. tuberculosis* pathways [7]; therefore, new drugs targeting alternative processes essential for *M. tuberculosis* to survive during infection are needed to expand the drug discovery portfolio.

An understanding of how *M. tuberculosis* adapts to its human host environments is key to finding more effective vaccines and therapeutic options. Central to pathogenesis is the interaction with the macrophage, where *M. tuberculosis* bacilli are able to survive and replicate intracellularly after phagocytosis [8]. As disease relies upon survival of *M. tuberculosis* within the macrophage, characterization of the mycobacterial adaptations to the macrophage intracellular environment highlights mechanisms of pathogenicity and identifies potentially druggable pathways and antigens to inform new anti-TB strategies. Genome-wide, unsupervised omics technologies have aided this approach, defining mycobacterial transcriptional signatures associated with an intracellular lifestyle [9,10,11,12,13], and identifying pathways essential for survival [14,15]. Studies of intracellular *M. bovis* BCG have also highlighted antigens most commonly presented by host macrophages [16].

Here, we define the transcriptional adaptations of *M. bovis* BCG, used for TB vaccination, to the human macrophage environment using RNA sequencing of intracellular bacteria. We compare the mycobacterial intracellular response to gene essentiality and antigen discovery datasets, highlighting potential candidate pathways for drug and vaccine development.

## 2. Materials and Methods

### 2.1. Mycobacterial Culture

*Mycobacterium bovis* BCG Montreal (ATCC 35735), containing pEGFP cloned under the control of mycobacterial 19 kDa promoter [16], was sub-cultured from frozen in Middlebrook 7H9 medium (Sigma, St. Louis, MO, USA) with 0.05% *v*/*v* tyloxapol supplemented with 10% oleic acid-albumin-dextrose-catalase (OADC) and 0.5% glycerol. Log phase bacteria for macrophage infections were harvested from 50 mL cultures in 250 mL vented Erlenmeyer flasks (Corning, Corning, NY, USA) after 3 days incubation at 37 °C with shaking (180 rpm). Log phase *M. bovis* BCG RNA was extracted after 5-day axenic culture (OD 0.35–0.46) from three independent biological replicates.

### 2.2. Macrophage Culture and Infection

Human monocyte THP-1 cells (ATCC:TIB-202) were maintained at 37 °C, 5% CO_2_ in RPMI medium supplemented with 2 mM L-glutamine, 10% *v*/*v* fetal bovine serum and 1 mM sodium pyruvate without antibiotics. THP-1 cells were chosen as a well-characterized immortalized cell line frequently used for mycobacterial research, where sufficient cell numbers could be reproducibly generated in a macrophage of human genetic background. Monocytes (1.1 × 10^6^ cells/mL) were differentiated into macrophage-like cells by 24 h stimulation with phorbol 12-myristate 13-acetate (20 nM final concentration). After washing twice with phosphate-buffered saline (PBS), cells were rested for 48 h in RPMI before infection. *M. bovis* BCG harvested from log phase culture was washed in PBS, resuspended in RPMI media, and syringed five times to generate a homogenous cell suspension before infecting macrophages at a multiplicity of infection of 10 bacilli: 1 macrophage for 4 h. After infection, cells were washed with sterile PBS to remove extracellular bacteria before incubation with fresh RPMI for 20 h at 37 °C in a 5% CO_2_ humidified incubator [16].

### 2.3. Mycobacterial RNA Extraction and RNA Sequencing

RNA was extracted from intracellular mycobacteria and from in vitro log phase *M. bovis* BCG using the GTC/TRIzol differential lysis method [11]. Mycobacterial RNA was DNase-treated and purified using the RNeasy spin-column system (Qiagen, Germantown, MD, USA). RNA quality and yield were evaluated using the Qubit Broad Range RNA assay (Thermo Scientific, Waltham, MA, USA), Nanodrop One (Thermo Scientific, Waltham, MA, USA) and Agilent 2100 Bioanalyzer (Agilent Technologies, Santa Clara, CA, USA). Three independent biological replicates of intracellular *M. bovis* BCG (2 µg/replicate) and 3 replicates of in vitro log phase *M. bovis* BCG RNA (2 µg/replicate) were depleted of mycobacterial rRNA using Ribo-Zero rRNA Removal (Bacteria) kit (Illumina, San Diego, CA, USA). Sequencing libraries were prepared using the NEBNext Ultra II kit (New England Biolabs, Ipswich, MA, USA) and paired-end sequenced using the Illumina NextSeq500 (75 × 2 paired end) instrument.

### 2.4. Transcriptomic Analyses

Paired-end raw sequence reads were initially processed with Trimmomatic (v0.36) [17] to remove low quality reads. The cleaned reads were mapped to the *M. bovis* AF2122/97 genome and to the human genome GRCh38 using Hisat2 [18], yielding 2–5 million reads mapped to the *M. bovis* genome per biological replicate. Gene expression was quantified using FeatureCounts from the Subread package v1.5.2. Differentially expressed genes in intracellular *M. bovis* BCG compared to log phase *M. bovis* BCG were identified using DESeq2 R package v.3.6.0, RLE normalized, and false discovery rates reduced using the Wald test with Benjamini and Hochberg multiple testing correction [19]. Reads mapping to multiple locations with the same number of mis-matched bases were ignored, and only primary alignments were counted. Genes with a log2-fold change (L2FC) <−1 or >1 with a corrected *p*-value < 0.05 were considered to be significantly differentially expressed (Table S1). To compare to the published literature, *M. bovis* AF2122/97 gene IDs were converted to *M. tuberculosis* H37Rv identifiers [20]. Significant overlaps with previously published transcriptional signatures, gene essentiality datasets and functional categories were identified using the hypergeometric function, *p*-value < 0.05. Differentially expressed genes were also mapped to *M. tuberculosis* metabolic pathways using gene set enrichment analysis [21] and DAVID Functional Annotation tool [22]. Fully annotated RNAseq data have been deposited in ArrayExpress; accession number E-MTAB- 11107.

## 3. Results

### 3.1. Mycobacterial Transcriptional Adaptations to Macrophage Infection

**Figure 1:**
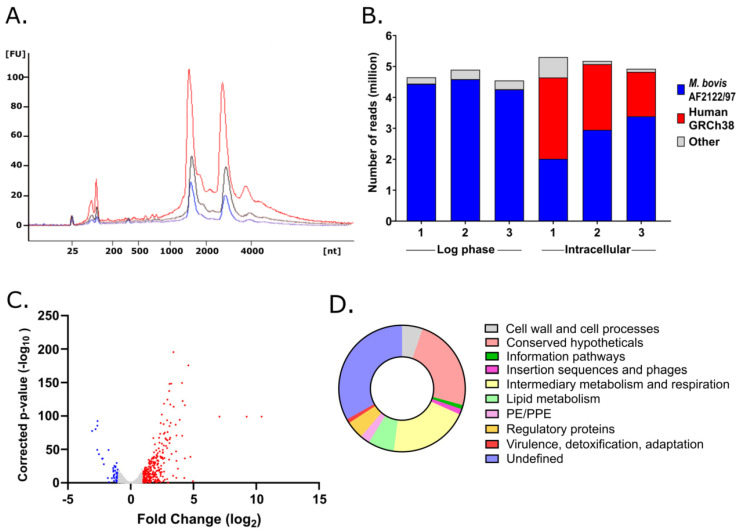
*M. bovis* BCG intracellular mRNA profile. (**A**). Bioanalyzer plot demonstrating size distribution of RNA extracted from infected THP-1 macrophages, showing defined bacterial ribosomal peaks of replicate extractions. Intracellular replicate 1 (black), 2 (blue), 3 (red). (**B**). Number of RNAseq reads mapped to *M. bovis* (blue) and human (red) genomes from each independent biological replicate. (**C**). Volcano plot identifying significantly differentially expressed genes comparing intracellular to log phase *M. bovis* BCG. Fold change (log2) >1 highlighted in red, <−1 highlighted in blue, non-significant in grey. (**D**). Distribution of functional categories represented by genes significantly induced in *M. bovis* BCG after macrophage infection (functional categories coloured clockwise).

To inform new treatment strategies for tuberculosis, we mapped the transcriptional adaptations of *M. bovis* BCG to the intracellular macrophage environment. Mycobacterial RNA was isolated from human THP-1 macrophages 24 h after infection. RNA, extracted using a differential lysis method (Figure 1A), was bacterial rRNA depleted and sequenced without further selection or amplification to maintain as representative a transcriptional signature as possible. RNAseq generated 2 to 5 million reads that aligned to the *M. bovis* AF2122/97 genome from each independent biological replicate (Figure 1B) [16].

**Figure 2:**
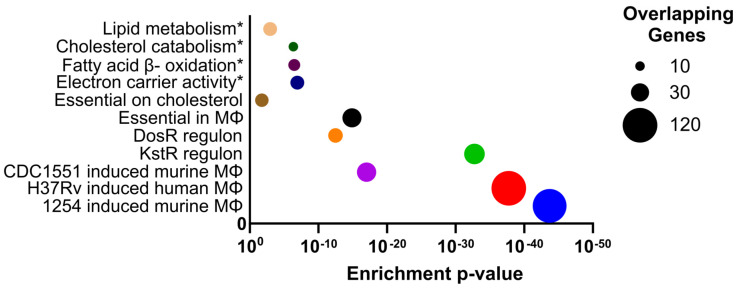
Features of the *M. bovis* BCG transcriptional response to the macrophage microenvironment highlighted by gene enrichment analyses of upregulated genes. Metabolic/respiratory pathways (marked with asterisks) identified from DAVID [22]; genes essential for growth on cholesterol [15], and for growth in macrophages [14]; regulons of DosR [23] and KstR [24]; and *M. tuberculosis* macrophage intracellular signatures with CDC1551 [10], H37Rv [11], and 1254 [9].

A total of 329 genes were significantly differentially expressed (L2FC >1/<−1, corrected *p*-value < 0.05), of which 284 were induced in intracellular *M. bovis* BCG and 45 genes were repressed in comparison to in vitro log phase bacilli (Figure 1C, Table S1). The two functional categories most represented in the upregulated genes (excluding ‘conserved hypotheticals’ and ‘undefined’) were ‘lipid metabolism’ and ‘intermediary metabolism and respiration’ (Figure 1D). Gene set enrichment analysis (GSEA) of induced genes supported these findings with the top functional cluster (enrichment score 5.6) involving fatty acid beta-oxidation using acyl-CoA dehydrogenase (*p* = 3.4 × 10^−7^), lipid metabolism (*p* = 4.5 × 10^−4^), and electron carrier activity (*p* = 1.2 × 10^−7^). The significance of altered carbon metabolism was further demonstrated by the second functional cluster (enrichment score 5.02) involving steroid metabolism (*p* = 1.1 × 10^−7^) and cholesterol catabolism (*p* = 4.6 × 10^−7^) (Figure 2). The *M. bovis* BCG intracellular signature overlapped closely with previously defined *M. tuberculosis* intracellular transcriptional profiles with changes to respiratory and metabolic pathways exemplified by induction of KstR and DosR regulons, upregulated to metabolise cholesterol and on exposure to hypoxia/nitric oxide, respectively, and upregulation of the iron-scavenging siderophore mycobactin biosynthetic genes (*mbtA*/*B*/*C*/*D*/*E*/*F*/*G*/*I*/*J*) (Figure 2). Accounting for these findings, we focused on conserved mycobacterial metabolic pathways with implications for drug and vaccine discovery pipelines.

### 3.2. Fatty Acid Metabolism and Cholesterol Catabolism

**Figure 3:**
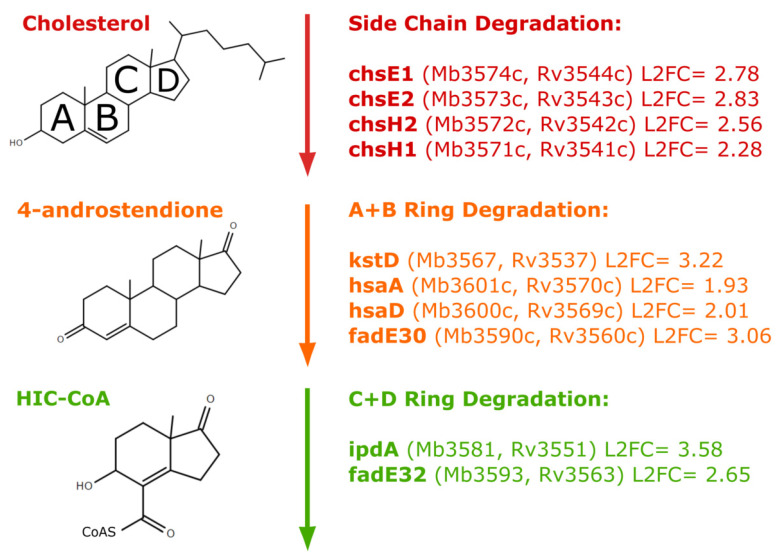
Cholesterol degradation pathway genes upregulated after macrophage infection. *M. bovis* BCG genes significantly induced intracellularly are marked on the cholesterol degradation pathway [27]. Gene name and log2 fold change (L2FC) values are detailed alongside *M. bovis* AF2122/97 and *M. tuberculosis* H37Rv identifiers.

*M. tuberculosis* inside macrophages metabolises fatty acids as a carbon source [25,26], and this is reflected in the induction of fatty acid metabolism genes intracellularly [9,12]. The key indicator gene *icl1* (*Mb0476*; *Rv0467*), involved in the glyoxylate cycle to convert acetyl-CoA molecules derived from the β-oxidation of fatty acids into oxaloacetate, was induced (L2FC 2.67) intracellularly by *M. bovis* BCG. In addition, β-oxidation of odd-chain fatty acids leads to the formation of propionyl-CoA, which cannot be used in the glyoxylate cycle. Instead, propionyl-CoA is converted to pyruvate via the methylcitrate cycle, a process requiring methylcitrate synthase and methylcitrate dehydratase, encoded by *prpC* and *prpD*, respectively (*Mb1162*, *Mb1161*; *Rv1131*, *Rv1130*). These were the most highly upregulated genes intracellularly (L2FC 9.24 and 10.42), further highlighting the significance of changing carbon metabolism in intracellular mycobacteria. Unsurprisingly, as identified by GSEA analysis, genes involved in cholesterol catabolism, many of which are regulated by KstR, were also induced (*chsE1*, *chsE2*, *chsH2*, *chsH1*, *hsaA*, *hsaD*) (Figure 3).

### 3.3. PE/PPE Family

**Figure 4:**
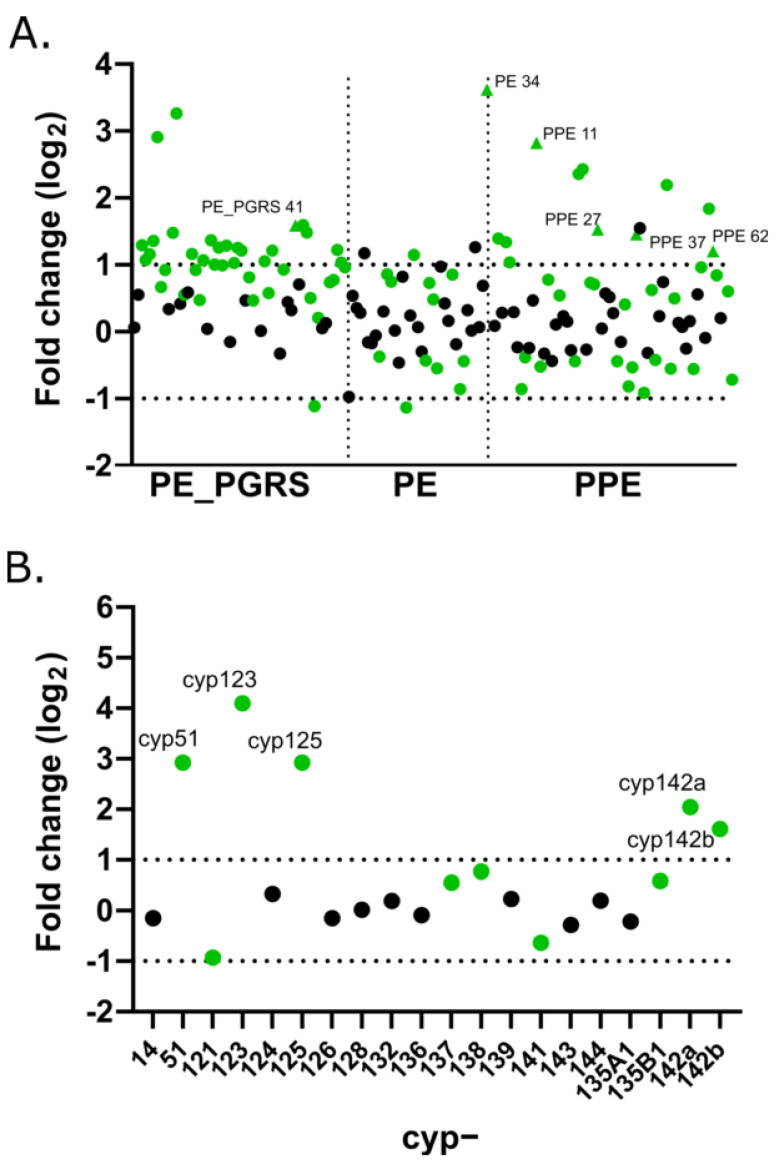
Intracellular gene expression levels of PE/PPE and cytochrome P450 families. Differential expression of the (**A**). PE/PPE/PE_PGRS families and (**B**). cytochrome P450 (*cyp*) family after 24 h macrophage infection compared to log phase in vitro bacilli. Triangles map to gene name labels in A. Green colour indicates corrected *p*-value < 0.05; horizontal dotted bars mark +1/−1 log2 fold change cutoffs.

PE/PPE encoding genes make up 10% of the mycobacterial genome coding capacity. These genes, with highly repetitive sequences, are less prevalent in non-pathogenic mycobacteria [28]. Of 157 *pe*/*ppe* genes, we found 35 to be significantly upregulated (L2FC > 1, *p* < 0.05) on macrophage infection (Figure 4a). Although the functions of many of these genes are unknown, *ppe11* (L2FC 2.82) and *pe34* (L2FC 3.62) have been linked to mycobacterial survival in the presence of lysozyme, hydrogen peroxide and acid stress, conditions that may be replicated in human macrophages [29,30]. Similarly, *ppe62* (L2FC 1.2) has been reported to be essential for iron acquisition [31]. Other *pe*/*ppe* genes, such as *ppe27* (L2FC 1.53), *ppe37* (L2FC1.46) and *pe-pgrs41* (L2FC 1.59), are implicated in manipulation of the host cell response [32].

### 3.4. Cytochrome P450 Family

CYP450 enzymes are a superfamily of heme containing enzymes involved in a range of intermediary metabolism and respiration pathways. There are 20 CYP450s identified in *M. tuberculosis* H37Rv (and *M. bovis* BCG) that likely function in diverse metabolic processes. Of these, *cyp51*, *cyp123*, *cyp125*, *cyp142a* and *cyp142b* were upregulated in macrophages (Figure 4b). In line with cholesterol catabolism genes that were upregulated, the products of *cyp125* and *cyp142* have been shown to oxidise cholesterol side chains as mycobacteria adapt carbon metabolism in the intracellular environment [33].

### 3.5. Overlap with Gene Essentiality Datasets

**Figure 5:**
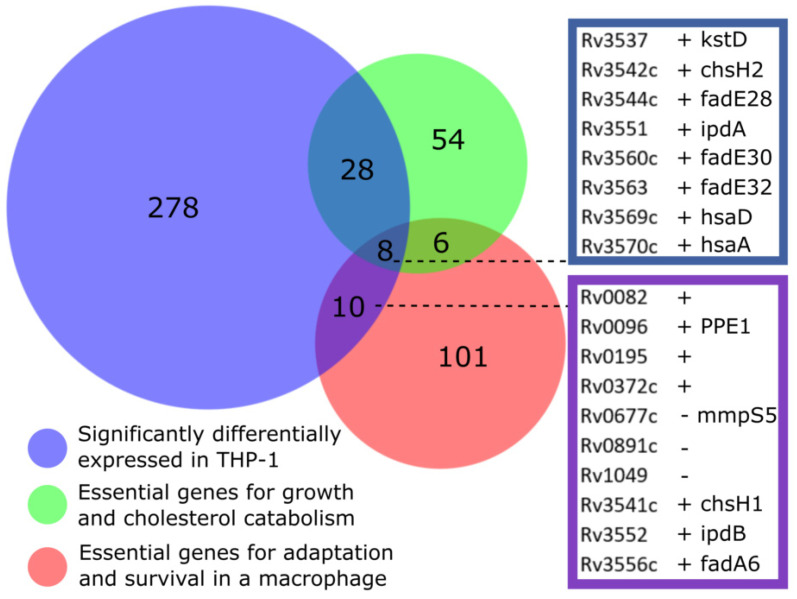
Overlap between transcriptional adaptations to macrophage infection and gene essentiality. Blue indicates the significantly differentially expressed genes after *M. bovis* BCG macrophage infection; green, the essential genes for growth on cholesterol [15]; red, the essential genes for macrophage infection [14]. Number of genes denoted in each section. Gene name and *M. tuberculosis* H37Rv identifiers marked for intersects; ‘+’ upregulated in macrophage, ‘-’ downregulated.

To highlight pathways for drug discovery that are both induced in the intracellular macrophage environment and essential for growth, we compared the *M. bovis* BCG intracellular transcriptional signature to genome-wide gene essentiality datasets. The most significant overlaps were with studies determining genes essential for cholesterol catabolism [15] (*p*-value = 1.3 × 10^−15^) and genes essential for growth in murine bone marrow derived macrophages [14] (*p*-value = 0.018) (Figure 2). Focusing on these two studies, we found that, from 324 genes (mapped to *M. tuberculosis* H37Rv) defining the transcriptional adaptations to macrophage phagocytosis, 28 genes were essential for growth and cholesterol catabolism [15], 10 genes for survival in the macrophage [14], and eight genes for both cholesterol and macrophage environments (Figure 5). As might be expected, these eight genes were all involved in cholesterol degradation (Figure 3). ChsE2 and ChsH2 have been linked to side chain degradation. KstD, HsaA and HsaD play roles in degradation of A and B rings, and FadE30 homologues in Actinomyces spp. have been shown to do the same. IpdA homologues in Actinomyces spp. drive the final stages of degradation, and FadE32 has been suggested to do the same [27].

Away from cholesterol metabolism, seven *M. bovis* BCG genes (Table 1) were induced on macrophage infection that were also essential for survival in murine macrophages [14], but not essential for cholesterol catabolism in vitro [15] (Figure 5). Upregulated *Rv0082* and *Rv3552*, encoding a probable oxidoreductase and possible CoA-transferase, respectively, are likely intermediate respiration and metabolism enzymes. *Rv0195* encodes a LuxR family regulator linked to mycobacterial growth recovery and survival of hypoxic and reductive stress [34]. Of the remaining genes, two are associated with lipid catabolism (*Rv3541c*, *Rv3556*), one is a member of the PPE family (*Rv0096*), and one encodes a conserved hypothetical protein of unknown function (*Rv0372c*). These genes are of interest in identifying potentially druggable pathways (separate from cholesterol catabolism) that are essential for macrophage survival and induced on infection. The functions of these genes are not fully elucidated and, therefore, warrant further investigation as potential therapeutic targets.

### 3.6. Comparison to the TB Vaccination Pipeline

Successful vaccination strategies target antigens that are expressed in vivo; therefore, we asked whether mapping the *M. bovis* BCG genes induced in macrophages might inform vaccine discovery efforts. There was no overlap between upregulated genes and antigens in vaccinations currently in clinical trials [35]. This is not surprising, as major vaccine candidate antigens such as ESAT-6 and CFP-10 are not present in *M. bovis* BCG. Intracellular growth had no significant impact on the expression of the other commonly included vaccine antigen, Ag85A.

The pre-clinical recombinant vaccine CMV-6Ag [36] includes the antigens Rv1733c, Rv2626c and Rv2389c. All three of the genes encoding these target proteins were significantly upregulated on macrophage infection (L2FC 1.46, 1.88, 2.19, respectively). The gene *hspX* (*Rv2031c*) was also induced (L2FC 4.28), the product of which is known to be highly immunogenic and an inducer of a strong Th1 immune response in mice [37]. Further comparison to 23 *M. tuberculosis* antigens, shown to be immunogenic through ELISA testing of TB patient blood IFN-γ responses [38], found that eight genes (five of which are regulated by DosR) were significantly induced (Table 2), and one gene (*Rv0440*) repressed by intracellular *M. bovis* BCG. The three most upregulated genes encoding immunogenic antigens from the Kassa et al. study were *Rv0079* (encoding a dormancy associated translation inhibitor), *rpfD* (coding for a resuscitation-promoting factor) and *fdxA* (involved in electron transfer). The remaining five genes encoded a transcriptional regulator of the response to hypoxia (Rv0081), a transmembrane protein (Rv1733c), a universal stress-associated protein (Rv2028c), and two conserved hypotheticals (Rv1734c, Rv2627). In addition, 14 mycobacterial genes induced intracellularly were also listed in the top 45 predicted vaccine candidate antigens selected by Zvi et al. [39] from comprehensive literature and *in silico* analyses (Table 2). These genes, the products of which have been determined to be immunogenic in TB patients or are predicted to be, and that show enhanced gene expression in the intracellular environment of a macrophage, should be prioritised for further examination.

## 4. Discussion

*M. tuberculosis* is able to survive and replicate in macrophages, and the initial host–pathogen interplay shapes subsequent pathogenesis [8]. The identification of metabolic pathways or immunogenic proteins important at this key stage of infection may offer new targets, and substantiate existing targets, for drug and vaccine discovery research. Here, we used differential RNA extraction methods alongside RNAseq to define the global transcriptional adaptations of *M. bovis* BCG, the TB vaccine, to human THP-1 macrophage infection. Aside from bacterial ribosomal RNA depletion and RNAseq library preparation, the RNA was not manipulated to retain a representative intracellular transcriptional signature. Good quality bacterial RNA, indicated by intact 16s and 23s ribosomal peaks, was isolated from three independent macrophage infections (Figure 1A). Degraded human macrophage RNA in the intracellular samples, denoted by minor 18s and 28s ribosomal peaks and spread of low/high molecular weight species, mapped to the human genome as expected. Two to five million reads per sample were mapped with high stringency to the *M. bovis* genome. This enabled the differential expression of highly repetitive gene sequences to be mapped with greater confidence, such as members of the PE/PPE gene family that are often ignored in genome-wide comparisons due to the repetitive nature of their sequences.

We used the vaccine strain *M. bovis* BCG Montreal containing a GFP plasmid in this study. *M. bovis* BCG shares 99% genome sequence similarity with virulent *M. tuberculosis*, there are also several important genomic modifications responsible for *M. bovis* BCG attenuation [40]. This includes Region of Difference 1 (RD1) encoding an ESX-1 system responsible for the secretion of CFP-10/ESAT-6 virulence factors [41]. Thus, macrophage infection models using *M. bovis* BCG are missing key pathogenic mechanisms of *M. tuberculosis* but capture the broad metabolic processes necessary for intracellular survival. Correspondingly, the 329 genes differentially expressed by *M. bovis* BCG intracellularly in this study overlap significantly with previously defined *M. tuberculosis* transcriptional adaptations to macrophage infection [9,10,11,12,42], featuring induction of glyoxylate shunt, methylcitrate cycle, cholesterol catabolism, and iron homeostasis signatures.

To highlight pathways for drug discovery that are important for mycobacterial survival in vivo and to avoid targets that become non-essential during infection [43], we compared the *M. bovis* BCG intracellular transcriptional response to genome-wide gene essentiality studies [14,15]. We found an overlap of 36 genes that were essential for growth with cholesterol and significantly induced after macrophage infection. This analysis underlines the importance of cholesterol catabolic pathways intracellularly, targeting drug discovery towards in vivo lifecycle stages of *M. tuberculosis*. This comparison also identified seven genes (Table 1) that were upregulated on macrophage infection and essential to survival in macrophages, but not linked to cholesterol gene essentiality. Many of these gene functions are unknown, and further investigation may highlight important roles in the adaptation of mycobacteria to the intracellular environment.

An alternative strategy to the above unsupervised approaches to identify potentially druggable pathways active in vivo is to explore the expression of gene families that likely have distinct roles across a diverse range of cellular processes. Such analyses for the highly repetitive PE/PPE family (*pe*, *ppe*, *pe*_*pgrs* gene families) and cytochrome P450s (*cyp* gene family) show induction of subsets of these gene families 24 h after macrophage infection (Figure 4), revealing novel pathways to target. Although the functions of many of these gene products are not well-understood, genes linked to survival under stress (*pe34*), iron acquisition (*ppe62*) and perturbation of the host response (*ppe27*, *ppe37*, *pe-pgrs41*) were highlighted as potentially important in vivo. Of the *cyp* genes induced intracellularly, enzymes encoded by *cyp125* and *cyp142* are involved in cholesterol catabolism and are suggested therapeutic targets, as inhibition of either of these enzymes leads to *M. tuberculosis* growth inhibition through cholest-4-en-3-one accumulation [44]. The sterol demethlyase encoded by *cyp51* is the target of azole antifungal drugs, such as econazole that is also effective against *M. tuberculosis*. The CYP450 enzymes, or the pathways that they operate in, identified by their upregulation after macrophage infection might make promising drug targets in *M. tuberculosis* [45].

To help define novel targets that are expressed during disease for vaccination strategies, we compared the genes upregulated by *M. bovis* BCG after macrophage infection with antigens used in subunit vaccines or found to be immunogenic in patients with TB [38] or predicted to be [39]. Three gene products (Rv1733c, Rv2626c and Rv2389c) overlapped with antigens in the preclinical CMV-6Ag subunit vaccine [36]. The products of 18 genes upregulated in macrophages were also demonstrated to be immunogenic in TB patients or predicted to be highly immunogenic (Table 2), suggesting that these may be potential targets for the development of future subunit vaccines.

In summary, we have characterized the *M. bovis* BCG response to human macrophage infection, generating RNAseq datasets for future investigations. The importance of β-oxidation of fatty acids, glyoxylate shunt, methylcitrate cycle, cholesterol catabolism and iron acquisition were reflected in the transcriptional adaptations to the intracellular environment. Overlaps with gene essentiality and antigen discovery studies highlight targets with potential for future anti-TB drug and vaccination strategies.

## Figures and Tables

**Table 1:** Genes induced by *M. bovis* BCG after macrophage infection that are also essential for growth in macrophages [14], but not essential for growth and cholesterol catabolism in vitro [15].

*M. bovis* Gene ID	H37Rv Gene ID	Gene Name	L2FC	Functional Category	Prediction of Function
Mb0085	Rv0082	-	2.4	Intermediary metabolism and respiration	Probable oxidoreductase, member of DosR regulon
Mb0099	Rv0096	ppe1	1.39	PE/PPE	PPE family protein
Mb0201	Rv0195	-	1.95	Regulatory	Possible transcriptional regulation
Mb0379c	Rv0372c	-	1.55	Conserved hypotheticals	Unknown
Mb3571c	Rv3541c	chsH1	2.28	Conserved hypotheticals	Cholesterol side chain degradation
Mb3582	Rv3552	ipdB	2.91	Intermediary metabolism and respiration	Possible CoA-transferase
Mb3586c	Rv3556	fadA6	2.04	Lipid Metabolism	Catalyses the formation of 4-methyl-5-oxo-octanedioyl-CoA in steroid catabolic pathway

**Table 2:** Genes induced by *M. bovis* BCG after macrophage infection relative to in vitro growth that were also demonstrated to be immunogenic in patients with active pulmonary tuberculosis [38] and/or are listed in the top 45 vaccine candidate antigens by Zvi et al. [39]. No asterisk = Kassa et al. [38] only; * = Zvi et al. [39] only; ** = identified in both studies.

*M. bovis* Gene ID	H37Rv Gene ID	Gene Name	L2FC	Functional Category	Prediction of Function
Mb0082	Rv0079 **	-	2.71	Conserved hypothetical	Dormancy associated translation inhibitor
Mb0084	Rv0081	-	2.09	Regulatory protein	Transcriptional regulatory protein, member of DosR regulon
Mb1762c	Rv1733c **	-	1.46	Cell wall and processes	Probable conserved transmembrane protein, member of DosR regulon
Mb1763c	Rv1734c	-	2.21	Conserved hypothetical	Unknown, member of DosR regulon
Mb2030c	Rv2007c	fdxA	2.15	Intermediary metabolism and respiration	Involved in electron transfer
Mb2053c	Rv2028c	-	1.21	Virulence, detoxification and adaptation	Universal stress protein, member of DosR regulon
Mb2660c	Rv2627c **	-	1.56	Conserved hypothetical	Unknown, member of DosR regulon
Mb2410c	Rv2389c **	rpfD	2.17	Cell wall and processes	Resuscitation-promoting factor
Mb1767	Rv1738 *	-	2.56	Conserved hypothetical	Implicated in control of non-replicating persistence
Mb2057c	Rv2031c *	hspX	4.28	Virulence, detoxification, adaptation	Heat shock protein, induced under stress
Mb2058	Rv2032 *	acg	1.01	Conserved hypothetical	Putative nitroreductase, induced under stress
Mb3154c	Rv3130c *	tgs1	2.47	Lipid metabolism	Triacylglycerol synthase
Mb3155	Rv3131 *	-	2.38	Conserved hypothetical	Putative nitroreductase
Mb1384	Rv1349 *	irtb	1.00	Cell wall and processes	Involved in iron homeostasis
Mb2054c	Rv2029c *	pfkB	1.30	Intermediary metabolism and respiration	Involved in glycolysis
Mb2055c	Rv2030c *	-	3.64	Conserved hypothetical	Unknown, induced under stress
Mb0476	Rv0467 *	icl	2.67	Intermediary metabolism and respiration	Involved in glyoxylate shunt
Mb1161	Rv1130 *	prpD	10.42	Intermediary metabolism and respiration	Involved in methylcitrate cycle

## Data Availability

Fully annotated RNAseq data have been deposited in ArrayExpress; accession number E-MTAB-11107.

## Supplementary Materials

The following supporting information can be downloaded at: https://www.mdpi.com/article/10.3390/vaccines10010113/s1, Supplementary Table S1: The *M. bovis* BCG response to THP-1 macrophage infection at 24 h. Differentially expressed genes in intracellular *M. bovis* BCG compared to log phase *M. bovis* BCG were identified using DESeq2 R package v.3.6.0, RLE normalized, and false discovery rates reduced using the Wald test with Benjamini and Hochberg multiple testing correction. Genes with a log2-fold change <−1 or >1 with an adjusted *p*-value < 0.05 were considered to be significantly differentially expressed. The table lists 329 differentially expressed genes, 284 induced and 45 repressed after macrophage infection relative to log phase in vitro growth. Table ordered by log2 fold change and *M. bovis* AF2122/97 identifier.
